# Impact of low-energy virtual monoenergetic imaging in photon-counting CT for pre-TAVI pelvic arteries visualization

**DOI:** 10.1007/s11547-025-02113-9

**Published:** 2025-10-06

**Authors:** Leona S. Alizadeh, Christian Booz, Thomas J. Vogl, Ludovica R. M. Lanzafame, Simon S. Martin, Ibrahim Yel, Leon D. Gruenewald, Vitali Koch, Tommaso D’Angelo, Silvio Mazziotti, Kerstin Smolka, Grit Braunegger, Daniel Dillinger, Leonhard Kaatsch, Daniel Overhoff, Niklas Verloh, Stephan S. Waldeck

**Affiliations:** 1 Department of Diagnostic and Interventional Radiology, University Hospital, Frankfurt, Germany; 2 Division of Experimental Imaging, Department of Diagnostic and Interventional Radiology, University Hospital, Frankfurt, Germany; 3 Diagnostic and Interventional Radiology Unit, BIOMORF Department, University Hospital Messina, 98124 Messina, Italy; 4 Department of Diagnostic and Interventional Radiology, Bundeswehr Central Hospital Koblenz, 56072 Koblenz, Germany; 5 Department of Vascular Surgery and Endovascular Surgery, Bundeswehr Central Hospital Koblenz, Rübenacher Straße 170, 56072 Koblenz, Germany; 6 Department of Diagnostic and Interventional Radiology, University Hospital Mannheim, 68167 Mannheim, Germany; 7 Department of Diagnostic and Interventional Radiology, Medical Center University of Freiburg, Faculty of Medicine, University of Freiburg, Freiburg, Germany; 8 Department of Diagnostic and Interventional Radiology, University Hospital Mainz, 55131 Mainz, Germany; 9 Diagnostic and Interventional Radiology Unit, BIOMORF Department, University Hospital “Policlinico G. Martino”, Via Consolare Valeria 1, 98100 Messina, Italy

**Keywords:** Photon-counting CT, Virtual monoenergetic Imaging, Pelvic arteries, Transfemoral access, Transcatheter aortic valve replacement

## Abstract

**Purpose:**

This study aimed to assess the impact of photon-counting computed tomography (PCCT) virtual monoenergetic images (VMI) on quantitative and qualitative parameters in abdominal and pelvic vascular imaging for transcatheter aortic valve implantation (TAVI) planning.

**Material and methods:**

A retrospective analysis of 125 patients undergoing dual-source PCCT scans before TAVI procedures was conducted. Reconstructions included polychromatic (T3D) images, leveraging multiple photon energy levels and VMI series spanning 40–100 keV in 15 keV increments. Quantitative parameters (signal-to-noise ratio [SNR] and contrast-to-noise ratio [CNR]) were evaluated. Qualitative assessments by three radiologists used clinically relevant five-point scales for overall image quality, TAVI access site suitability, and confidence in TAVI measurements.

**Results:**

VMI reconstructions, particularly at 40 and 55 keV, demonstrated significantly higher SNR and CNR than T3D reconstructions (*p* < 0.001). T3D reconstructions had a mean noise of 12.61 ± 6.12, comparable to 100 keV VMI reconstructions (14.77 ± 8.23, *p* > 0.05). In qualitative evaluation, 55 keV VMI images scored highest in overall image quality and TAVI access site assessability, followed by 70 keV VMI reconstructions.

**Conclusion:**

Low-keV PCCT VMI reconstructions provided superior quantitative and qualitative image quality for abdominal and pelvic vascular imaging in TAVI planning. Notably, 55 keV reconstructions showed an image quality reserve over T3D images, aiding confidence in TAVI-related measurements and enabling possible future reductions in contrast agent use, emphasizing the relevance of VMI techniques in optimizing TAVI imaging protocols.

## Introduction

The most recent significant development in the fast-evolving field of computed tomography (CT) is the emergence of clinical photon-counting computed tomography (PCCT) [1–11]. In addition to high spatial resolution, PCCT allows spectral data to be extracted from any spectral postprocessing (SPP) dataset, enabling the analysis and differentiation of energy-dependent changes in attenuation of different materials, surpassing the capabilities of the latest-generation of source and detector-based dual-energy CT (DECT) technology [7].

In this context, virtual monoenergetic imaging (VMI) is a technique that allows for the reconstruction of monoenergetic images at specific keV energy levels [1, 12–16]. In vascular imaging, VMI has been used to optimize image quality through material and vascular plaque decomposition, enhancement of vascular contrast and contrast-to-noise ratios (CNR) for accurate stenosis evaluation, and assessment of dissections [17–21]. So far, preliminary DECT and PCCT studies on vascular imaging have shown that low VMI reconstructions can provide superior image quality and CNR values compared to standard polychromatic 120 kV series (termed T3D in PCCT), such as the study from 2022 by Dillinger et al. on abdominal PCCT [4, 22]. PCCT facilitates the application of these advanced imaging techniques to routine clinical examinations [11].

To date, the use of VMI in transcatheter aortic valve implantation (TAVI) planning has been infrequently explored. Martin et al. assessed VMI with third-generation dual-source DECT in 2017, and Rippel et al. compared thoracoabdominal photon-counting CT with energy-integrating detector CT in general, focusing explicitly on cohorts with low-contrast attenuation [23, 24]. According to reports from Germany, the annual rate of TAVI procedures was 22.973 in 2019, underlining the fundamental role and importance of this life-saving medical intervention in cardiovascular medicine [25]. TAVI procedures are commonly performed on patients who show a range of comorbidities, including severe cardiovascular diseases, diabetes, hypertension, chronic kidney disease, and respiratory disorders, among others [26]. Therefore, TAVI procedures can be more challenging than other intravascular procedures [25, 27, 28].

Pre-procedural CTA scans, therefore, play a pivotal role in TAVI procedure planning, determination of suitable sites for vascular access, and implementation of the commonly used long 14–16 F sheaths, requiring a minimum cross-diameter of the vessels of 5–6 mm in the pelvic axis [29–31]. However, to our knowledge, no studies have been conducted so far evaluating PCCT low-keV VMI specifically for pelvic artery assessment in the context of transfemoral access planning for TAVI. This work uniquely explores the optimization of low-keV VMI reconstructions to improve diagnostic image quality and planning confidence. Thus, we aimed to assess the impact of low-keV VMI PCCT reconstructions on quantitative and qualitative image quality, vascular contrast, and diagnostic assessability of the pelvic arteries in patients undergoing PCCT examinations before TAVI.

## Materials and methods

### Study design

**Fig. 1:**
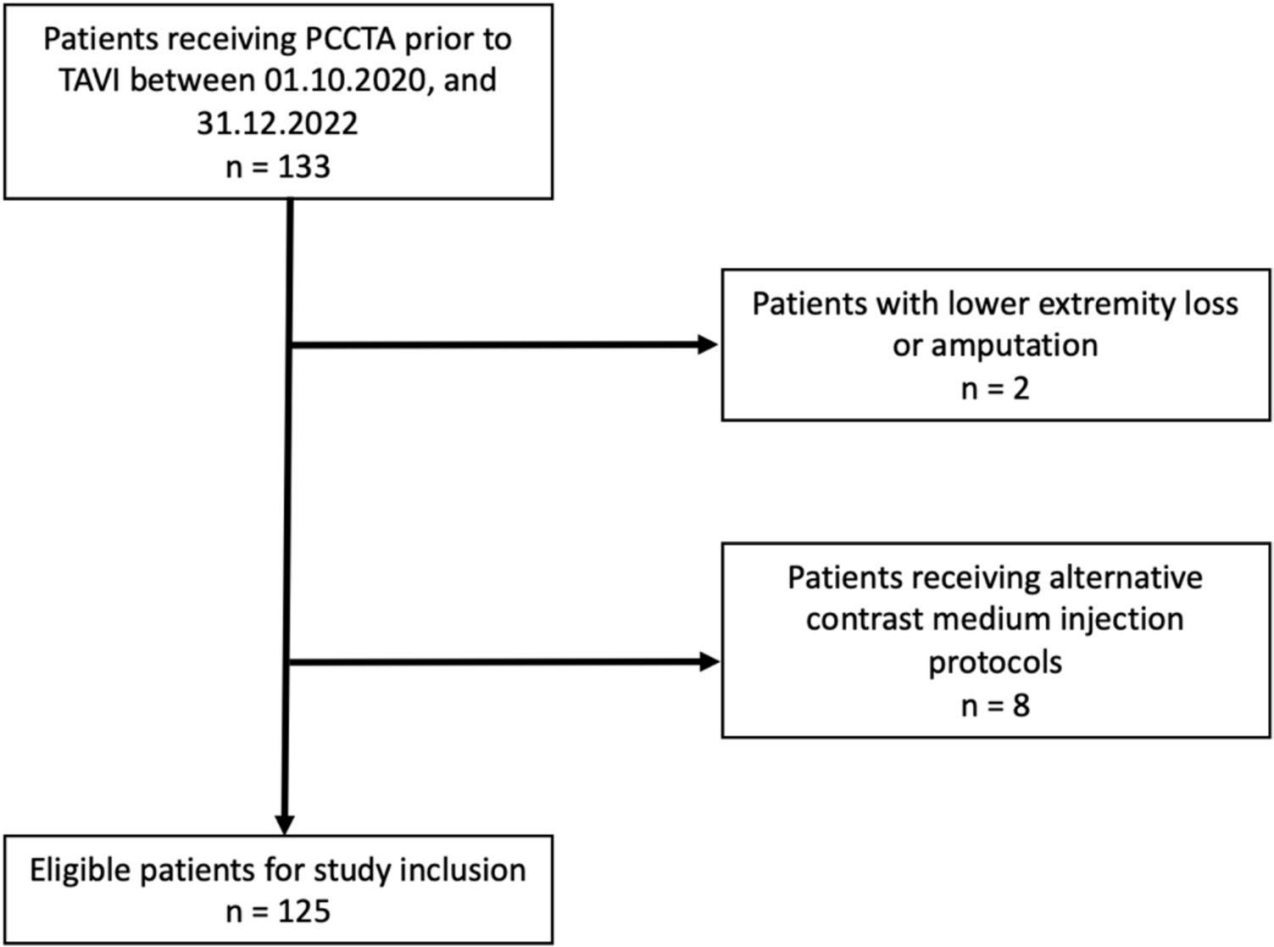
Flowchart visualizes this study’s patient inclusion process and the amount and reasons for patient exclusion. PCCTA: Photon-Counting CT angiography

This study was conducted in accordance with the Declaration of Helsinki in a retrospective, single-center format and received approval from the local institutional review board of the University Hospital Frankfurt with a waiver of informed consent. No funding was received. Patients were included as follows: we initiated a search in our PCCT database for patients who underwent clinically mandated CTA studies for runoff examinations before TAVI between October 2020 and July 2023. All patients from this period were then included consecutively. We excluded any patients with above-ankle amputations or lower extremity loss and acquisitions deviating from the standard PCCTA protocol or contrast medium injection technique. The study inclusion process is displayed in Fig. 1.

### CTA scan protocol

All image data were acquired utilizing a first-generation dual-source photon-counting CT (PCCT) system (Naeotom Alpha; Siemens Healthineers, Forchheim, Germany) operating in full spectral imaging mode. The scan was conducted in a craniocaudal direction, involving an ECG-synchronized CT angiography (CTA) covering the region from the skull base to the proximal segment of the superficial femoral artery. Vascular enhancement was achieved through an automated power injector CT Motion (Ulrich Medical, Ulm, Germany), delivering a total of 80 mL of iodinated contrast material in a single bolus (Xenetix 350, 76.78 g/100 mL Iobitridol, Guerbet, Roissy, France) at a rate of 3.5 mL/s, followed by a bolus of 50 mL of saline. This administration was executed using a 20-gauge needle inserted into a superficial vein in the antecubital fossa. The angiographic phase was started bolus triggered (region of interest in the descending aorta). The start of the acquisition was chosen automatically to ensure data acquisition of the aortic valve at a 40% RR interval [32].

Patients were positioned supine, head first. Initial anterior–posterior and lateral localizer scout images were obtained after initial patient positioning, facilitating a modern ceiling-mounted camera-guided system.

Data collection was executed with the following parameters: pitch of 3.2, slice thickness of 1 mm, increment of 0.7 mm; collimation of 2 × 144 × 0.4 mm for both detectors; a field of view (FOV) of 350 mm; and tube voltage was automatically switched between 100 and 120 kV, with the inclusion of a tin filter.

These tube settings are the vendor-specific defaults for the first-generation PCCT system. Automated real-time anatomical tube current modulation (CareDose 4D; Siemens) was activated as a default setting.

### Spectral monoenergetic CTA image reconstruction

Raw data from PCCT was reconstructed utilizing a soft tissue convolution kernel (Qr40; Siemens) with a section thickness of 1.0 mm. Iterative reconstruction was performed using the Quantum Iterative Reconstruction (QIR) 3 algorithm.

Subsequently, the image data was transferred to a multimodality workstation (syngo.via version VA70; Siemens) and processed in the dedicated spectral imaging application (CT VMI; Siemens). The VMI image series was reconstructed at energy levels varying from 40 to 100 keV in 15-keV increments. VMI energy levels above 100 keV were not considered in this study, as iodine attenuation at higher energies is reported to fall below diagnostic thresholds for CTA [33]. For reference, polychromatic T3D reconstructions were generated by integrating all detected photon energies.

### Quantitative analysis

The VMI mode facilitated by PCCT enables a single ROI measurement within the monoenergetic data set to derive values for all accessible VMI levels. Despite this, we performed an average of three measurements for each evaluation to ensure data consistency. These measurements were carried out by an experienced radiologist with twelve years of vascular CT imaging and research experience. Hounsfield units and standard deviation values were noted for each measurement.

The 8-segment model previously described by Martin et al. [23] was adapted to focus on the key segments relevant to pelvic vascular anatomy and used for standardized vessel segmentation, with ROIs placed in the following vessel segments: supraceliac abdominal aorta (1), distal aorta 2 cm proximal to the aortic bifurcation (2), right and left common iliac artery (3 + 4), right and left common femoral artery (5 + 6), right and left superficial femoral artery (7 + 8). All measurements were performed by setting an ROI as large as possible without including vessel borders, calcifications, noncalcified plaque, or stent material. The average ROI sizes were 166 ± 25 mm^2^ for aortic segments and 34 ± 8 mm^2^ for iliofemoral segments. In vessels with an implanted stent, measurements were taken proximal to the stent. The ROI was placed proximal to the vasculopathy in vessel segments with partial occlusion or prominent vascular plaques. Completely occluded vessel segments or those with severe stenosis, calcifications, or motion artifacts were excluded from the objective image quality analysis. Subcutaneous fat and the psoas muscle densities were used for contrast assessments. Image noise was defined by the standard deviation of Hounsfield Units in the retroperitoneal fat. The signal-to-noise ratio (SNR) and contrast-to-noise ratio (CNR) were calculated using the following formulas.

$${\text{SNR }} = {\text{ HUartery}}/{\mathrm{SDfat}}$$

$${\mathrm{CNR}} = \left( {{\mathrm{HUartery}} - {\mathrm{HUmuscle}}} \right)/{\mathrm{SDfat}}$$

### Qualitative analysis

We performed two independent subjective image quality assessments based on previous research and clinical experience on DECT and VMI. First, for the overall image quality score, the degree of vascular contrast attenuation and visible image noise levels were rated using an established image-scoring system, as described by Yuan et al. in *Radiology in 2012* [34]. Second, a rating of a score for the evaluation of TAVI access site assessability was performed based on clinically relevant criteria described in the literature for effective TAVI planning, such as meticulous access site assessment, pelvic artery diameter measurements, and usefulness of the images for pre-interventional procedure planning [23, 35]. This assessment included an evaluation of the iliofemoral arteries for the transfemoral approach and assessing vessel sizing for TAVI device compatibility and diagnostic confidence for detecting contraindications (dissection, thromboembolism, rupture, bleeding, etc.). Three independent readers with 5, 7, and 15 years of CTA experience and two years of PCCT angiography (PCCTA) experience analyzed images reconstructed at energy levels from 40 to 100 keV in 15 keV steps, using the two 5-point Likert scales and clinically relevant parameters for pre-TAVI vascular PCCTA (compare to Table 1 for further information). The readers were blinded to the image reconstruction technique and assessed each image series individually in a randomized order, with the possibility to individually change the windowing from the standard soft tissue window. To mitigate recall bias, a single randomly selected image series (40–100 keV VMI and T3D) from each patient was evaluated per reading session, maintaining a week's gap between sessions.

**Table 1:** A five-point scoring system for evaluation of image quality and assessability of Transcatheter Aortic Valve Implantation (TAVI) planning parameters from Photon-Counting Computed Tomography virtual monoenergetic image reconstructions

Scale and score	Overall image quality and vascular contrast	Assessability of transfemoral access site
1	Poor quality, poor opacification	Nondiagnostic, true lumen not delineable, measurements not possible
2	Suboptimal opacification	True lumen visibility and measurements with low confidence and suboptimal visibility
3	Acceptable opacification	Sufficient confidence in measurements and acceptable visibility of the true lumen
4	Good opacification	High confidence in measurements and good visibility of the true lumen
5	Excellent, no visible noise, excellent opacification	Excellent confidence in measurements, excellent visibility of the true lumen

"Overall Image Quality and Vascular Contrast" score indicates opacification and image clarity. In contrast, the "Assessability of Transfemoral Access Site and TAVI-related measurements" score describes visibility and confidence in the TAVI-related vessel measurements. Scores range from 1 (poor/nondiagnostic) to 5 (excellent)

### Radiation dose

To estimate the radiation dose in PCCTA, the volume CT dose index (CTDI) and dose-length product (DLP) for each patient were extracted from structured report (SR) data. The effective dose was then computed using the DLP and conversion factors (0.015 mSv/mGy∙cm) as recently delineated in the publication by Kopp et al. [36]; these factors are derived from the International Commission on Radiological Protection (ICRP) Publication 103 [37]. The DLP, automatically calculated by the CT scanner, was multiplied by the conversion factor specific to the abdomen and pelvis to ascertain the effective dose of this region [36].

### Statistical analysis

Statistical computations were performed using R software (R Foundation for Statistical Computing, Vienna, Austria). Descriptive statistics for continuous variables were calculated, including the mean, standard error of the mean, median, standard deviation, minimum, and maximum values. All values were tested for normality using the Kolmogorov–Smirnov test. For the comparative analysis, we used a two-way analysis of variance (ANOVA) for ranks. Friedman's test examined differences in objective image quality between the T3D images and between different VMI reconstructions at each keV level. For the subjective image analysis comparison, the average final 5-point score from the readers was compared across the three algorithms using Friedman's test.

The degree of agreement on subjective image quality scores was calculated using intraclass correlation coefficients (ICCs) in a two-way mixed-effects model. ICC values were interpreted as follows: values of 0.81 or higher were deemed excellent, 0.61 to 0.80 good, 0.41 to 0.60 moderate, 0.21 to 0.40 fair, and below 0.20 poor. A p-value less than 0.05 was considered a statistically significant difference.

## Results

### Patient demographics

The final cohort consisted of 65 males, aged 65.9 ± 11.8 years (range 58–84 years), and 60 females, aged 61.7 ± 8.1 years (range 61–79 years), undergoing PCCT before TAVI at our institution. The patients’ mean body mass index (BMI) was 27.4 ± 6.3 kg/m^2^, ranging from 18.5 to 31.7 kg/m^2^. The mean heart rate was 73.3 ± 15.3 beats per minute, ranging from 48 to 122 beats per minute.

### Quantitative analysis

**Fig. 2:**
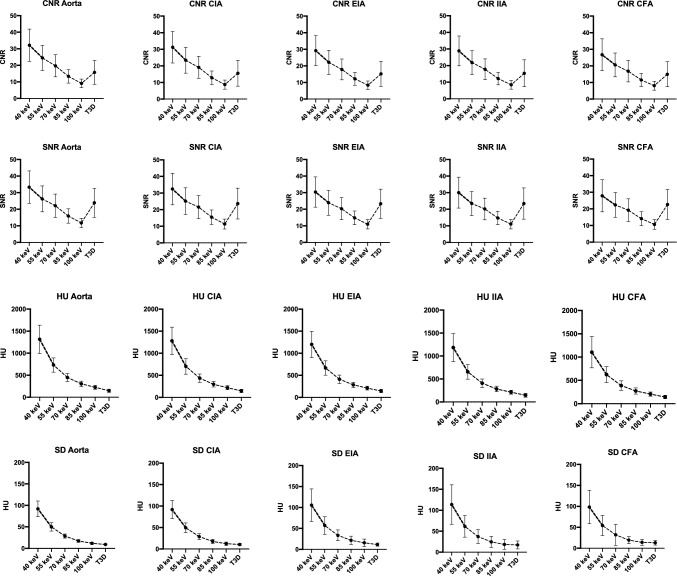
Mean plot illustrating the quantitative image quality measurements at different energy levels. The measurements include the mean attenuation (HU) and image noise (SD), as well as signal-to-noise ratio (SNR) and contrast-to-noise ratio (CNR) at 40, 55, 70, 85, 100 keV and T3D reconstructions (polychromatic reconstruction), calculated for five distinct arterial regions: the aorta, common iliac artery (CIA), external iliac artery (EIA), internal iliac artery (IIA), and common femoral artery (CFA). Each data point represents the mean value, and error bars denote the standard deviation

The average attenuation, measured in Hounsfield units (HU), decreased consistently with increasing energy levels from 40 to 100 keV for all examined arteries. The highest mean attenuation was recorded at 40 keV in the aorta (1315.40 ± 320.54 HU) and the lowest in the CFA at T3D series (142.57 ± 37.44 HU) (compared to T3D imaging, low-energy VMI reconstructions from 40 to 100 keV showed increased attenuation values in all investigated regions (all p < 0.001, except for 100 keV vs T3D p = 0.074). Exemplary values of the CFA at different VMI reconstructions were: 1104.72 ± 335.62 HU for 40 keV, 624.31 ± 171.80 for 55 keV, 389.19 ± 101.30 HU for 70 keV, 271.85 ± 65.35 HU for 85 keV, and 205.61 ± 45.47 HU for 100 keV versus 142.57 ± 37.44 HU for T3D reconstructions (*p*-values: 40–70 keV vs T3D *p* < 0.001, 85 keV vs T3D *p* = 0.003, 100 keV vs T3D *p* = 0.126) (compare to Fig. 2 and Table 2 for complete results of all regions’ attenuation assessments).

**Table 2:** Results of the quantitative measurements in the aorta, common iliac artery (CIA), external iliac artery (EIA), internal iliac artery (IIA), and common femoral arteria (CFA)

Parameters	40 keV VMI	55 keV VMI	70 keV VMI	85 keV VMI	100 keV VMI	T3D	*p*-values
Attenuation (HU)	1206.10 ± 319.82	669.69 ± 169.98	415.28 ± 97.86	286.19 ± 63.01	213.43 ± 44.66	144.97 ± 37.88	All comparisons, *p* < 0.001
Aorta	1315.40 ± 320.54	732.06 ± 161.26	449.40 ± 93.80	305.03 ± 60.17	225.07 ± 42.02	148.51 ± 36.50	All comparisons, *p* < 0.001
CIA	1280.20 ± 308.83	702.01 ± 175.93	435.02 ± 96.30	296.58 ± 62.99	218.56 ± 45.74	145.82 ± 38.58	All comparisons, p < 0.001
EIA	1200.01 ± 296.26	665.86 ± 161.88	411.47 ± 94.82	283.92 ± 60.88	211.12 ± 43.78	144.41 ± 38.38	All comparisons, *p* < 0.001
IIA	1184.39 ± 303.99	655.14 ± 159.72	408.39 ± 92.75	282.92 ± 60.54	212.51 ± 43.46	145.29 ± 41.40	All comparisons, *p* < 0.001
CFA	1104.72 ± 335.62	624.31 ± 171.80	389.19 ± 101.30	271.85 ± 65.35	205.61 ± 45.47	142.57 ± 37.44	All comparisons, *p* < 0.001
Noise	101.17 ± 37.12	54.97 ± 20.74	32.39 ± 15.94	20.42 ± 9.41	14.77 ± 8.23	12.61 ± 6.12	All comparisons, *p* < 0.001 except 70 keV vs. T3D *p* = 0.004, 85 keV vs. T3D *p* = 0.404, 100 keV vs T3D p = 0.838 85 keV vs. 100 keV *p* = 0.030
SNR	30.53 ± 9.60	24.01 ± 7.67	20.52 ± 6.81	14.91 ± 4.24	11.09 ± 2.92	23.34 ± 8.89	All comparisons, *p* < 0.001 except 55 keV vs. T3D *p* = 0.988, 70 keV vs. T3D p = 0.059
Aorta	33.33 ± 9.78	26.24 ± 7.81	22.14 ± 7.03	15.94 ± 4.32	11.71 ± 2.87	23.89 ± 8.75	All comparisons, *p* < 0.001 except 55 keV vs T3D *p* = 0.506, 70 keV vs. T3D p = 0.501
CIA	32.42 ± 9.52	25.15 ± 7.99	21.55 ± 6.93	15.46 ± 4.36	11.36 ± 3.00	23.67 ± 9.27	All comparisons, *p* < 0.001 except 55 keV vs. T3D *p* = 0.350, 70 keV vs T3D *p* = 0.334
EIA	30.38 ± 9.09	23.92 ± 7.53	20.31 ± 6.61	14.79 ± 4.15	10.96 ± 2.83	23.31 ± 8.83	All comparisons, *p* < 0.001 except 55 keV vs. T3D *p* = 0.685, 70 keV vs T3D *p* = 0.093
IIA	30.03 ± 9.33	23.48 ± 7.23	20.17 ± 6.52	14.71 ± 4.05	11.05 ± 2.90	23.45 ± 9.43	All comparisons, *p* < 0.001 except 55 keV vs. T3D *p* = 0.988, 70 keV vs T3D *p* = 0.059
CFA	27.89 ± 9.68	22.38 ± 7.43	19.24 ± 6.82	14.14 ± 4.22	10.66 ± 2.93	22.65 ± 8.98	All comparisons, *p* < 0.001 except 40 keV vs. T3D *p* = 0.003, 55 keV vs. T3D *p* = 0.876, 70 keV vs T3D p = 0.065
CNR	29.35 ± 9.49	22.27 ± 7.46	18.09 ± 6.49	12.32 ± 3.98	8.47 ± 2.68	15.32 ± 7.53	All comparisons, *p* < 0.001 except 70 keV vs. T3D p = 0.097, 85 keV vs T3D *p* = 0.058
Aorta	32.15 ± 9.74	24.48 ± 7.55	19.71 ± 6.65	13.34 ± 4.04	9.09 ± 2.60	15.79 ± 7.25	All comparisons, *p* < 0.001 except 70 keV vs. T3D *p* = 0.140, 85 keV vs T3D p = 0.263, 100 keV vs. T3D p = 0.007
CIA	31.24 ± 9.46	23.39 ± 7.75	19.11 ± 6.58	12.87 ± 4.09	8.74 ± 2.76	15.56 ± 7.80	All comparisons, *p* < 0.001 except 70 keV vs. T3D *p* = 0.042, 85 keV vs T3D p = 0.080
EIA	29.20 ± 9.03	22.16 ± 7.26	17.87 ± 6.26	12.20 ± 3.87	8.34 ± 2.59	15.21 ± 7.51	All comparisons, *p* < 0.001 except 70 keV vs. T3D p = 0.077, 85 keV vs T3D p = 0.077
IIA	28.84 ± 8.96	21.81 ± 7.18	17.76 ± 6.28	12.18 ± 3.81	8.44 ± 2.63	15.35 ± 8.11	All comparisons, *p* < 0.001 except 70 keV vs. T3D *p* = 0.083, 85 keV vs T3D p = 0.059
CFA	26.71 ± 9.63	20.62 ± 7.17	16.80 ± 6.49	11.54 ± 3.97	8.05 ± 2.69	14.95 ± 7.56	All comparisons, *p* < 0.001 except 70 keV vs. T3D p = 0.222, 85 keV vs T3D *p* = 0.048

All data are displayed in mean ± standard deviation (SD). HU: Hounsfield unit; SNR: signal-to-noise ratio; CNR: contrast-to-noise ratio; VMI: Virtual monoenergetic images

Image noise demonstrated a consistent decrease with increasing energy levels. At 40 keV, the noise value was 101.17 ± 37.12, while at 100 keV, it was significantly less at 14.77 ± 8.23 (*p* < 0.001). Compared to 100 keV results, T3D imaging presented a mean noise of 12.61 ± 6.12, resulting in a *p*-value of *p* = 0.838 in the one-way ANOVA test.

The SNR values gradually decreased with energy level increments from 40 to 100 keV for all arterial regions, with the highest value at 40 keV for the aorta (33.33 ± 9.78) and the lowest at 100 keV for the CFA (10.66 ± 2.93) (all *p* < 0.001). T3D imaging showed average SNR values of 23.34 ± 8.89, close to the 55 keV SNR results of 24.01 ± 7.67 (p = 0.368).

Lastly, CNR values exhibited a similar trend to SNR, decreasing from 40 to 100 keV. The highest CNR was found at 40 keV for the aorta (32.15 ± 9.74), and the lowest at 100 keV for the CFA (8.05 ± 2.69) (*p* < 0.001). T3D imaging displayed a relatively lower overall mean CNR of 15.32 ± 7.53. The one-way ANOVA revealed significant differences between each region of interest (*p* < 0.001). Further post hoc analysis indicated that each group significantly differed in a pairwise comparison (*p* < 0.001). Complete results from the quantitative image analysis are consolidated in Table 2 and visually represented in Fig. 2.

### Qualitative analysis

Qualitative analysis revealed the highest ratings for the 55 keV VMI reconstructions, with an overall image quality and vascular contrast rating of 4.6 ± 1.1 and an evaluation of TAVI access site assessability rating of 4.7 ± 0.7. This category had a good interrater agreement, with an ICC of 0.64 (95% CI 0.39–0.77). This result was followed closely by the 70 keV VMI reconstructions, with an overall image quality and vascular contrast rating of 4.5 ± 0.8 and an assessability rating of 4.4 ± 1.0 (ICC, 0.63; 95% CI 0.42–0.81; *p* = 0.31), as well as the 40 keV VMI reconstructions, with an overall image quality and vascular contrast rating of 4.1 ± 0.8 and an assessability rating of 4.2 ± 1.2 (ICC, 0.66; 95% CI 0.42–0.81; *p* = 0.014). T3D reconstructions achieved scores of 4.0 ± 1.2 for overall image quality and 3.9 ± 0.9 (ICC, 0.61; 95% CI 0.52–0.73), demonstrating good interrater agreement (*p* < 0.001 for 40–85 keV).

**Fig. 3:**
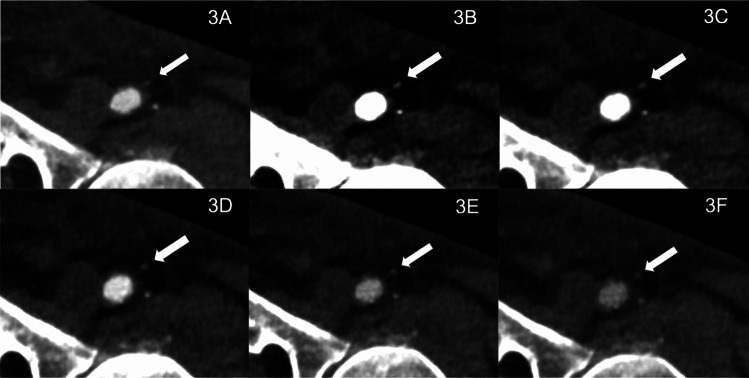
Photon-counting CT angiography in a 65-year-old male with aortic valve stenosis Pre- Transcatheter Aortic Valve Implantation (TAVI): Utilizing 75 ml of 320 mg/mL iodixanol (Visipaque, GE Healthcare) at 3.5 ml/second. Displayed are T3D reconstructions (**A**) and monoenergetic 40–100 keV reconstructions in 15 keV intervals (**B**–**F**) of the left common femoral artery (CFA). T3D (polychromatic) reconstructions showed no vascular pathology. Low-keV reconstructions provided an increase in CNR, peaking at 40 keV VMI (virtual monoenergetic imaging). Optimal image quality for interpretation was noted at 55–70 keV. A smaller artery superficial to the CFA (arrow), potentially at risk during TAVI sheath insertion, was masked at T3D but could clearly be delineated in 40 and 55 keV VMI reconstructions, underscoring the need for careful navigation to avoid inguinal hemorrhage, a common TAVI complication

**Fig. 4:**
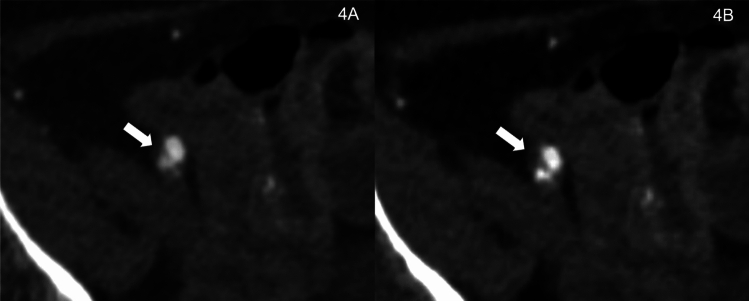
Photon-Counting CT angiography of a 68-year-old Male with diabetes and hypertension Pre- Transcatheter Aortic Valve Implantation (TAVI): Displayed are polychromatic T3D (**A**) and monoenergetic 55 keV reconstructions (**B**) of the right common femoral artery (CFA). This patient, with no history of vascular interventions, underwent PCCTA using 75 ml of 320 mg/mL iodixanol (Visipaque, GE Healthcare) at 3.5 ml/second. The T3D reconstruction of the right CFA showed a modest decrease in intraluminal iodine concentration. In contrast, the low-keV monoenergetic reconstruction at 55 keV highlighted the true vessel lumen and an intimal dissection membrane – information crucial for assessing transfemoral access and TAVI-related parameters

**Fig. 5:**
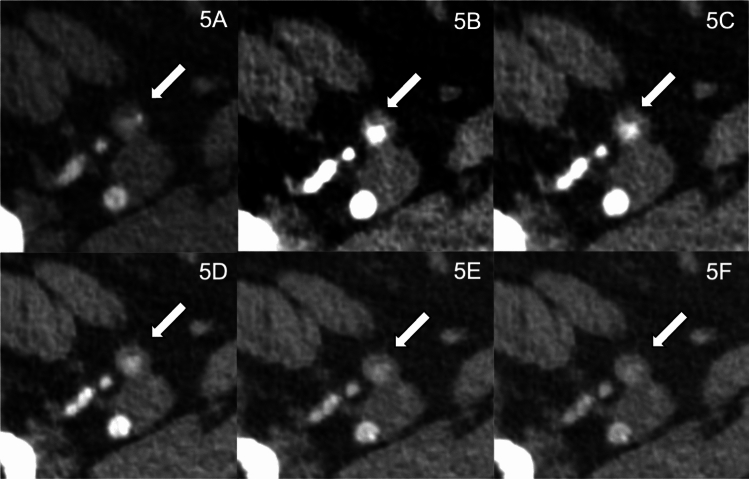
Photon-Counting CT angiography (PCCTA) in a 74-Year-Old Female with Multiple Comorbidities Pre- Transcatheter Aortic Valve Implantation (TAVI): Displayed are polychromatic T3D reconstructions (**A**) and monoenergetic 40–100 keV reconstructions in 15 keV intervals (**B**–**F**) of the right superficial femoral artery (SFA). This patient, who has diabetes, aortic valve stenosis, renal impairment, and NYHA class III heart failure, underwent PCCTA using 75 ml of 320 mg/mL iodixanol (Visipaque, GE Healthcare) at 3.5 ml/second. Without a history of vascular interventions, the T3D polychromatic reconstructions revealed reduced intravascular iodine contrast in both proximal SFAs, more pronounced on the right side. The monoenergetic reconstructions detailed the true lumen diameter and significant asymmetrical wall thickening with positive hypodense intimal remodeling of the right SFA. While the T3D images might indicate chronic subtotal occlusion, the 40 keV and 55 keV VMI reconstructions showed reduced yet present flow, suggesting a need for potential intervention to avert progression and subsequent acute limb ischemia. The residual lumen diameter in these images also indicated the infeasibility of sheath insertion on the right side for the TAVI procedure

Figures 3, 4, and 5 demonstrate the clinical value of low-keV reconstructions’ higher overall image quality in pre-interventional PCCT.

When comparing across the range of energy levels, VMI series from 40 to 85 keV received superior overall image quality ratings (all *p* < 0.01) compared to those from 100 keV and T3D images.

The results of the subjective image analysis are summarized in Table 3 and depicted visually in Fig. 3. Overall, the global ICC score for all three observers was 0.56 (95% CI 0.41–0.68), indicating moderate interrater agreement.

**Table 3:** Results of the qualitative image ratings using a Likert Scale for Virtual Monoenergetic Imaging (VMI) and polychromatic T3D Reconstructions

Energy Level (keV)	Image Rating (Overall Image Quality and Vascular Contrast)	Image Rating (Assessability of Transfemoral Access Site)	Intraclass Correlation Coefficient (ICC)
40	4.1 ± 0.8	4.2 ± 1.2	0.66 (95% CI 0.42–0.81)
55	4.6 ± 1.1	4.7 ± 0.7	0.64 (95% CI 0.39–0.77)
70	4.5 ± 0.8	4.4 ± 1.0	0.63 (95% CI 0.42–0.81)
85	4.2 ± 1.1	4.1 ± 0.7	0.54 (95% CI 0.36–0.67)
100	3.5 ± 0.9	3.2 ± 1.3	0.52 (95% CI 0.33–0.61)
T3D	4.0 ± 1.2	3.9 ± 0.9	0.61 (95% CI 0.52–0.73)

Mean ratings ± standard deviation are shown for energy levels ranging from 40 to 100 keV in VMI and a separate category for polychromatic T3D reconstructions. CI: Confidence interval

### Radiation dose

All included examinations adhered to the examination protocols without complications, and no examination in this cohort had to be repeated. The mean cumulative CT dose index (CTDIvol) across all examinations was 6.14 ± 3.83 mGy. The mean cumulative dose-length product (DLP) was recorded as 412.45 ± 248.34 mGy*cm.

## Discussion

In the existing literature, several publications can be found that focus on cardiovascular and aortic PCCT, or standard T3D image evaluation for TAVI planning, with only a few focusing on low-keV VMI and access site planning. Therefore, we aimed to test if low-keV VMI PCCT can improve TAVI and access planning, with a focus on pelvic vessels, in clinical routine scans [1, 4, 10, 11, 33, 38, 39]. As a key finding, the quantitative image quality analysis of low-keV PCCT VMI reconstructions showed higher vascular attenuation compared to T3D images between 40 and 70 keV, with the maximum HU, CNR, and SNR values at 40 keV, while also showing the highest image noise in these reconstructions. Our findings can be explained by an attenuation peak near the k-edge value of iodine at 33.17 keV, leading to the highest values in both SNR and CNR calculations [12, 29, 33]. Regarding qualitative image quality, radiologists preferred low-keV VMI reconstructions in the 55–70 keV range over those at 40 keV and the higher 85–100 keV range, including traditional T3D reconstructions. The 55 keV reconstructions received the highest ratings for an overall image quality score of 4.6 ± 1.1 and a transfemoral access site assessability score of 4.7 ± 0.7. Close behind, the 70 keV reconstructions scored 4.5 ± 0.8 for overall image quality and 4.4 ± 1.0 for transfemoral access site assessability, with no significant difference in performance (*p* = 0.31). Despite scoring highest in quantitative analysis, 40 keV reconstructions were ranked lower in qualitative assessments. This discrepancy is probably due to significant image noise and exaggerated blooming artifacts at energy levels below 55 keV. These obscure vessel boundaries and particularly exaggerate calcified plaques, often leading to overestimation of stenosis. Additionally, low-keV imaging can potentially mask soft plaques and lead to an underestimation of noncalcified narrowing. Subtle findings, such as dissection membranes (see Fig. 4), could be masked, ultimately reducing diagnostic confidence in diseased or heavily calcified vessels. These results underline the trade-off between signal intensity and interpretability at lower keV values. Interestingly, a recent investigation by Dirrichs et al. comparing standard non-spectral DECT and T3D PCCT reconstructions of the aorta for TAVI planning resulted in significantly higher scores for visual image quality in PCCT of 4.8 ± 0.4 vs. 3.3 ± 0.6 in DECT [39]. In our investigation, we showed that low-keV VMI expands this image quality reserve further, as our 55 keV rating scores (4.6 and 4.7) significantly surpassed the results of the T3D images (4.0 and 3.9) for TAVI planning, underlining the potential of optimized PCCT reconstructions for clinical use. Literature underscores the importance of clear visualization of pelvic arteries and optimized procedure planning for transfemoral access and intravascular navigation in TAVI procedures. Considering the high risk of complications associated with the prevalent vascular pathologies in this patient group, aiming for the highest possible diagnostic image quality is crucial [25, 26, 40]. In a direct comparison with the third-generation dual-source DECT VMI + results from Martin et al., our PCCT findings demonstrated marginally higher SNR and CNR values across all measured regions. Specifically, the maximum SNR value for PCCT was 33.33 ± 9.78 at 40 keV (DECT, 27.8 ± 13). For CNR, PCCT results peaked at 32.15 ± 9.74 at 40 keV (DECT, 26.3 ± 12.7). In terms of maximum HU, our study observed a peak at 1315.40 ± 320.54 HU for 40 keV PCCT VMI reconstructions of the abdominal aorta, which is notably higher than the DECT maximum of 971.9 ± 115.5 HU reported in the same region by Martin et al.. These results suggest that while VMI reconstructions in PCCT follow a similar attenuation pattern as seen in low-keV images of the latest-generation DECT, they may exhibit superior CNR and SNR values with reduced image noise. However, it should be noted that differences in scanner technology, acquisition settings, and reconstruction protocols between PCCT and DECT directly influence image quality and quantitative measurements, limiting the direct comparability of results. Regarding qualitative image quality, our optimal ratings at 55 keV align closely with the findings by Martin et al., where the best scores were obtained at 50–60 keV for DECT [23].

While lower keV imaging with PCCT can provide high CNR values, it is crucial to balance these quantitative advantages with the qualitative aspects of image interpretation [13, 19, 23]. The best clinical outcomes will likely be achieved by optimizing imaging parameters to balance high CNR and acceptable noise levels, considering the radiologists’ preferences for the best image quality.

This points toward the potential advantages of utilizing PCCT low-keV VMI for advanced pre-TAVI imaging and transfemoral access planning, offering a promising avenue for refining current clinical practice. While DECT low-keV VMI requires acquiring a specific DECT protocol, PCCT facilitates VMI reconstructions of any dataset if SPP data acquisition is enabled, representing the standard setting for the system used in this study. As it is not uncommon for patients to not have images from prior diagnostic examinations, the decision to use a DECT protocol can be challenging, especially for inexperienced radiologists and radiological technicians.

This not only underlines the benefits of using PCCT compared to DECT in terms of mere hardware aspects, such as the higher spatial resolution compared to a latest-generation DECT system, but also PCCT improves practicability aspects of implementing spectral imaging in clinical everyday routine scans [7, 10, 41].

The ability to generate images at different energy levels enables radiologists to identify and characterize soft tissue structures, assessing the size, shape, and anatomical characteristics of the aorta and pelvic vessels and visualizing vascular pathologies such as dissections, with higher clarity and contrast [13, 16, 18, 23, 33, 42, 43].

Figures 3 and 4 are cases highlighting the benefits of VMI for procedure planning by visualization of small arteries in the access site (Fig. 3) or even a small dissection membrane (Fig. 4), which may cause bleeding or a need for vascular intervention later on.

By optimizing CNR and enhancing vascular contrast, VMI may help to delineate vessel boundaries more accurately, enabling precise measurements of vessel dimensions. This information is crucial for selecting the appropriate size and positioning of the transcatheter valve during TAVI planning [17, 27, 35, 44].

Moreover, VMI offers the advantage of reducing the need for contrast agents, as it enhances the intrinsic contrast of the vascular structures, as recently described by Higashigaito et al. and others [45–47]. This may potentially minimize the risk of contrast-related adverse events by facilitating a reduction in contrast volume, particularly in patients with impaired renal function [6, 48–51]. This feature is particularly beneficial in visualizing poorly contrasted vessels, a common issue in TAVI candidates due to low systemic cardiac output fraction or systemic diseases such as diabetes [33]. In the context of the spectral resolution of VMI, PCCT excels over DECT, as DECT does not possess the same spectral discrimination capabilities, rendering PCCT a superior modality for imaging pre-TAVI patients [7].

Several study limitations should be acknowledged. First, since this was a retrospective study, the VMI reconstructions were not used to guide or influence procedural planning or access site decisions. Future prospective studies could investigate this clinical impact directly. Secondly, the manual measurement of the ROI could have been compromised in some instances by vessel disease, particularly when dealing with small vessel diameters or residual lumens affected by calcifications and soft plaques. Variability in quantitative vessel measurements between readers was not analyzed. Thirdly, the choice of energy levels, which ranged from 40 to 100 keV with 15 keV increments, was made arbitrarily, and therefore other energy levels should be tested in future studies. Lastly, our study did not explore higher energy levels. This decision was based on our specific focus on the advantages of VMI at lower energy levels for improving iodine signal and image quality in pre-TAVI CTA imaging [29]. Further, blooming artifacts were not quantitatively analyzed in this study, while it is known that calcium load might be exaggerated under low-keV imaging due to high X-ray attenuation of calcified plaque [29, 52]. This limitation should be considered in future research. Despite these limitations, the advantages of integrating VMI PCCT into clinical routine for TAVI planning are indisputable. The notable VMI capabilities of PCCT enable personalized image reconstructions and enhanced vascular contrast, ultimately improving diagnostic image quality by extracting additional information across various keV levels. This improved diagnostic confidence during pre-TAVI imaging enables PCCT to enhance patient safety and may contribute to higher procedural success rates.

In conclusion, low-keV VMI PCCT reconstructions demonstrated superior quantitative and qualitative image quality results, along with increased vascular contrast and diagnostic assessability scores in pre-TAVI imaging. In the clinical pre-TAVI setting, the high spectral resolution and improved CNR and SNR values displayed significant advantages over conventional CT imaging for the detection and assessment of vascular pathologies. As more evidence from larger and multi-vendor studies emerges—and experience with this technology grows—VMI PCCT may become a standard in pre-TAVI imaging.

## Data Availability

The data will not be provided for publication at the moment due to further upcoming research in our department.

## Abbreviations

AA: Abdominal aorta
BMI: Body mass index
CFA: Common femoral artery
CIA: Common iliac artery
CNR: Contrast-to-noise ratio
CT: Computed tomography
CTA: CT angiography
CTDI: Computed tomography dose index
DECT: Dual-energy CT
DLP: Dose-length-product
EIA: External iliac artery
ICRP: International Commission on Radiological Protection
IIA: Internal iliac artery
keV: Kilo-electron-volt
PCCT: Photon-counting computed tomography
PCCTA: Photon-counting computed tomography angiography
SFA: Superior femoral artery
SPP: Spectral postprocessing
SR: Structured report
TAVI: Transcatheter aortic valve implantation
T3D: 120 kV polychromatic image reconstruction
VMI: Virtual monoenergetic imaging
